# Determination of the optimal enrichment *Artemia franciscana* with a synbiotic combination of probiotics *Pediococcus acidilactici* and prebiotic fructooligosaccharide

**Published:** 2017-03-15

**Affiliations:** 1 *Department of Fishery, Faculty of Natural Resources, Urmia University, Urmia, Iran; *; 2 *Department of Food Hygiene and Quality Control, Faculty of Veterinary Medicine, Urmia University, Urmia, Iran.*

**Keywords:** *Artemia franciscana*, Enrichment, Fructooligosaccharide, *Pediococcus acidilactici*, Synbiotic

## Abstract

In this study the optimal amount of enriching *Artemia franciscana* with a synbiotic combination of *Pediococcus acidilactici* and fructooligosaccharide (FOS( was investigated. The experiment was conducted in a completely randomized design in ten treatments: multi-level probiotics *P. acidilactici* (1×10^9^, 1×10^8^ and 1× 10^7^ CFU per L) and multi-level enriched prebiotic FOS (1, 2 and 5) g per L of solution and control. To evaluate the enrichment of adult artemia with each treatment, sampling was performed at 2, 4 and 6 hr after initiation of enrichment. The results showed that a synbiotic containing a probiotic treatments *P. acidilactici* at 1 × 10^9^ and 1 × 10^8^ CFU per L had more bacteria than a synbiotic containing a probiotic treatment with 1 × 10^7 ^CFU per L (*p *< 0.05), but did not show significant difference between levels of 1 × 10^9 ^and 1 × 10^8^ CFU per L (*p *> 0.05). The highest number of attached bacteria (6.78 ± 0.03 log CFU g^-^1) to adult artemia was shown after 6 hr of enrichment that showed significant difference with 2 hr, but did not show significant difference with 4 hr time. In conclusion, the results of this study showed that adult artemia in a short time (about 4 hr) unlike nauplii artemia can retain a large amount of probiotic (1 × 10^8^ CFU *P. acidilactici* per L and 5 g per L FOS prebiotic) on their own.

## Introduction

In recent years the use of probiotics in aquaculture has become prevalent and can overcome many of the problems associated with bacterial diseases.^1^ Various types of microalgae (*Tetraselmis*), yeasts (*Phaffia, Saccharomyces*), Gram-positive bacteria (*Bacillus, Carno-bacterium, Enterococcus, Lactobacillus, Lactococcus, Micrococcus, Streptococcus* and *Weissella*) and Gram-negative bacteria (*Aeromonas, Alteromonas, Photorhodo-bacterium, Pseudomonas* and *Vibrio*) have been studied as probiotics.^2^ The ambiguities in the use of probiotics such as the non-guaranteed viability of the probiotics in the gastrointestinal tract, necessity of competition of introduced probiotic with commensal microbiota and the ability to form the mass as well as the long-term sustainability of the masses, caused the researchers to gain the idea of prebiotic.^3^^,^^4^

The prebiotics are selectively fermented by potentially beneficial bacteria groups (e.g LAB) and result in increased numbers and dominance of these beneficial bacteria in the intestinal tract.^5^^,^^6^ Combined administration of probiotics species with appropriate prebiotics (synbiotic) as a substrate to increase dominance and sustainable growth of probiotics bacteria has been suggested due to the inability of probiotic species to form stable masses and maintain dominance in the gut microbiota.^7^

Regarding the use of synbiotics in aquaculture nutrition, there are relatively limited studies regarding administration of synbiotics in aquaculture. However, the results of those studies revealed positive effects on physiology and immunity.^8^^-^^11^

Artemia has been widely used in aquaculture due to the high nutritional value, the proper size and the possibility of enrichment.^12^ It can be used as the carrier of particles used in aquaculture such as nutrients (fatty acids, vitamins, etc.), antimicrobial substances, vaccines and probiotics.^13^ Administration of live, beneficial and non-pathogenic bacteria in the culture medium or artemia culture can have positive effects on cultured fish species via improvment in the intestinal microbiota, eliminating harmful bacteria and improving the nutritional value of artemia.^14^^,^^15^ The number of bacteria in the artemia is exponentially increased at the time of artemia hatching and enrichment processes by nutrients.^16^ It also has been observed that in the early stages of development in fish larvae, the increase in the number of bacteria in the intestinal microflora of fish is mainly associated with the bacteria in live food.^17^ It can be concluded that with increase in the number of opportunistic bacteria in the fish intestine, mortality becomes more in the intensive culture of early life stages of fish and control of bacterial population in the live feed may lead to higher survival rates of fish larvae and profitability in hatcheries.^18^

However, the use of synbiotic in the early life stages of fish through the enrichment of live food and it’s the effects on growth, physiology and immunity has not been studied, yet. The use of synbiotic in artemia could be considered as a food for artemia, and also could affect intestinal flora, the immune system and increase resistance to pathogenic bacteria, enhance health and reduce the risk of disease outbreaks and fry mortality. The present study was conducted because the literature is poor regarding the optimal enrichment *A. franciscana* with a synbiotic for use in fish larvae and hatcheries.

## Materials and Methods

**Artemia culture conditions and Bacterial strain. **Artemia (*A. franciscana*) cysts were obtained from Great Salt Company, Utah, USA. Chorionic layer of cysts were separated by the use of sodium hypochlorite during decapsulation. Hatching of decapsulated cysts was performed through the use of cone-shaped container with a volume of 120 L and sea water (salinity of 30 g per L). Cysts were incubated with a density of 5 g per L at 30 ˚C with 2000 lux lighting conditions and vigorous aeration.^19^

Artemia nauplii were transferred to culture environment after hatching. The culture environment was cone-shaped plastic containers (150 L) aerated by aeration pipes connected to the central pump. Nauplii were fed during the first few days by spirulina algae (*Spirulina platensis*) powder, and thereafter fed with a mixture of rice bran, baker's yeast and spirulina. Feeding was performed three times a day with an interval of 4 hr. Stocking density was three nauplii per mL and culture period was 20 days to reach sexual maturity.^20^ During this time, all physical and chemical parameters were measured and recorded daily. Physiochemical factors such as water temperature, salinity, dissolved oxygen, light and pH during culture period were monitored and maintained at 28.69 ˚C, 32 g L^-1^, 7.75 mg L^-1^, 1500 lux and 7.88, respectively.

Commercial probiotic used in this experiment was prepared from Tak Gene Company Pediguard^®^ Tehran, Iran) contains 1 × 10^10 ^CFU g^-1 ^*Pediococcus acidilactici*. Prebiotic, fructooligosaccharide (FOS; Raftilose P95) was supplied from Orafti Company, Oreye, Belgium.

**Enrichment of synbiotic to artemia adult. **For enrichment of adult artemia with the number of 4000 in each treatment (the average total length 4.51 ± 0.28 mm and the mean individual weight was 2.59 ± 0.09 mg) by synbiotic, combinations of probiotics and prebiotics were used in accordance with the Table 1. Thus, for the suspension preparation, first a ratio of 0.1:10 lecithin and water at 40 ˚C were poured into a clean and dry beaker and were mixed using an electric mixer. Then the rapeseed oil was added to this solution and was mixed very well by mixer. The ratio of lecithin, colza oil and water in suspension was 0.1, 1 and 10, respectively. To evaluate the diameter of oil particle, some samples were poured on slide and were observed under light microscope. The prepared suspension (150 mL), probiotic *Pediococcus acidilactici* and prebiotic, FOS were transferred to the beaker and were uniformed with an electric mixer, then mix in 2 L of seawater and adult artemia with the number of 4000 was placed inside the container (Table 1).^4^^,^^21^

**Table 1:** Adult artemia enrichment levels and different treatments

**Treatments**	**Probiotics ** ***P. acidilactic*** **i (CFU L** ^-1^ **)**	**Prebiotic ** **FOS ** **(g L** ^-1^ **)**
**Control (T1)**	0	0
**Synbiotics (T2)**	1 × 10^9^	1
**Synbiotics (T3)**	1 × 10^9^	2
**Synbiotics (T4)**	1 × 10^9^	5
**Synbiotics (T5)**	1 × 10^8^	1
**Synbiotics (T6)**	1 × 10^8^	2
**Synbiotics (T7)**	1 × 10^8^	5
**Synbiotics (T8)**	1 × 10^7^	1
**Synbiotics (T9)**	1 × 10^7^	2
**Synbiotics (T10)**	1 × 10^7^	5

FOS: Fructooligosaccharide.

In all treatments 150 mL rapeseed oil was separated from prepared suspension and probiotic and prebiotic were transferred in 2 L of seawater.

**Artemia adult microbiology. **To examine the process of enrichment, sampling was performed from the all treatment after the start of enrichment, 2, 4 and 6 hr after enrichment.^22^ Amount of 100 mL (containing 0.5 g of adult artemia) were collected using a sterile pipette in each of the mentioned time and were transferred to a filter with a mesh size of 300 µm, then to eliminate bacteria in the external surface of artemia body, were washed for 60 sec in a salt solution, Benzalkonium chloride (0.1 %) (BIC Graphic, Indianapolis, USA) and again were washed with sterile water and after that, water of samples was taken after a while.^17^ The sterile samples were weighted and transferred to sterile porcelain mortar. After the homo-genization of samples using a sterile saline solution (0.87 % w/v), dilutions of 10^-1^ to 10^-7^ were prepared. From prepared dilutions, under sterile conditions, the volume of 0.1 mm was removed and was transmitted to de Man, Rogosa and Sharpe (MRS) medium (Merck, Darmstadt, Germany) to determine the number of lactic acid bacteria and was spread on surface of the plate. The incubation of plates was conducted for 3 to 5 days in an incubator at a temperature of 30 ˚C and under aerobic conditions. After the incubation period, the bacteria were counted, and recorded according to the logarithm of the colony unit (the number of bacterial colonies grown on culture medium × dilution coefficient^-1^) per g of artemia.^23^ *Pediococcus acidilactici* was investigated and identified based on apparent characteristics, gram staining and also some standard biochemical tests such as phenol red, citrate, indole, motion and methyl red.^24^ Colony forming units per gram of artemia were determined for viable bacterial populations. 16S rRNA partial sequence analysis was used to confirm identification of *P. acidilactici* isolates as described by Merrifield *et al*.^14^

**Statistical analysis. **After checking the normality data and homogeneity of variance, two-way ANOVA followed by Duncan's multiple range tests was used for data analysis. Mean values were considered significantly different at *p* < 0.05. Statistical analyses were conducted using SPSS statistical package (version 21.0; SPSS Inc., Chicago, USA). 

## Results

The effects of different treatments and different times of the amount of bacteria present in the enriched artemia are shown in Table 2. The results indicated that probiotic bacteria in each of the enrichment time, were successfully enriched inside artemia. The enrichment trend of *Artemia franciscana* at different times used in this experiment was different. In terms of the enrichment time, the results showed the capability of artemia enrichment had significant difference (*p* < 0.05). Regarding the synbiotic and probiotic treatments at 4 and 6 hr after the start of enrichment, there was no significant difference in the number of attached bacteria per gram of artemia (*p* > 0.05).

**Table 2:** Counting the number of bacteria (*Pediococcus acidilactici*) enriched artemia treatments *in vivo* (log CFU g^-1^). Data are presented as mean ± standard error

**Treatment**	**Time of enriched**		
	**2 hr**	**4 hr**	**6 hr**
**Control (T1)**	1.09 ± 0.05^a^	1.23 ± 0.04^a^	1.15 ± 0.04^a^
**Synbiotics (T2)**	5.67 ± 0.07^c^	6.71 ± 0.03^d^	6.78 ± 0.07^d^
**Synbiotics (T3)**	5.58 ± 0.04^c^	6.67 ± 0.05^d^	6.50 ± 0.05^d^
**Synbiotics (T4)**	5.73 ± 0.07^c^	6.78 ± 0.04^d^	6.81 ± 0.04^d^
**Synbiotics (T5)**	5.45 ± 0.03^c^	6.31 ± 0.07^d^	6.50 ± 0.12^d^
**Synbiotics (T6)**	5.50 ± 0.07^c^	6.61 ± 0.07^d^	6.71 ± 0.04^d^
**Synbiotics (T7)**	5.30 ± 0.07^c^	6.50 ± 0.11^d^	6.23 ± 0.05^d^
**Synbiotics (T8)**	4.81 ± 0.02^b^	5.68 ± 0.08^c^	5.71 ± 0.04^c^
**Synbiotics (T9)**	4.60 ± 0.04^b^	5.61 ± 0.05^c^	5.65 ± 0.07^c^
**Synbiotics (T10)**	4.78 ± 0.03^b^	5.50 ± 0.04^c^	5.21 ± 0.04^c^

abcd Same letters indicate no significant difference between the groups (*p* > 0.05).

**Fig. 1:**
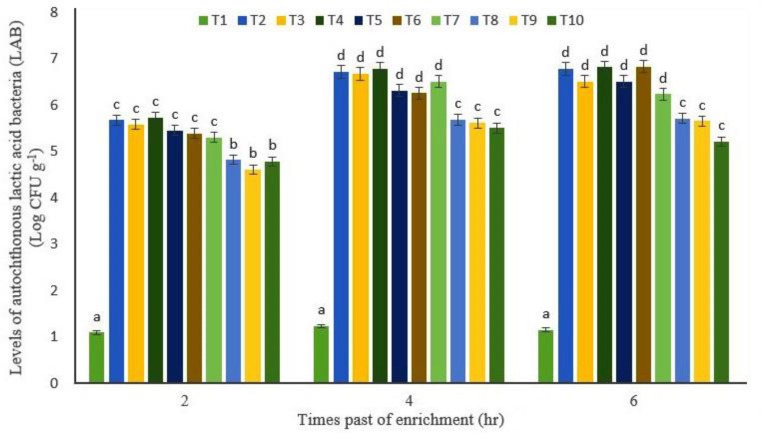
Levels of autochthonous lactic acid bacteria (Log CFU g^-1^) of adult artemia at different times in different treatments including: T1) Control, T2) Synbiotic (1g L^-1 ^FOS prebiotic and , 1 × 10^9^ CFU.L^-1^ *P. acidilactici *probiotic, T3) Synbiotic (2g L^-1 ^FOS prebiotic and 1 × 10^9^ CFU L^-1^ *P. acidilactici *probiotic, T4) Synbiotic (5 g L^-1 ^FOS prebiotic and 1 × 10^9^ CFU L^-1^ *P. acidilactici *probiotic, T5) Synbiotic (1 g L^-1 ^FOS prebiotic and 1 × 10^8^ CFU L^-1^ *P. acidilactici *probiotic, T6) Synbiotic (2 g L^-1 ^FOS prebiotic and 1 × 10^8^ CFU L^-1^ *P. acidilactici *probiotic, T7) Synbiotic (5 g L^-1 ^FOS prebiotic and 1 × 10^8^ CFU L^-1^ *P. acidilactici *probiotic, T8) Synbiotic (1 g L^-1 ^FOS prebiotic and 1 × 10^7^ CFU L^-1^ *P. acidilactici *probiotic, T9) Synbiotic (2 g L^-1 ^FOS prebiotic and 1 × 10^7^ CFU L^-1^ *P. acidilactici *probiotic, and T10) Synbiotic (5 g L^-1 ^FOS prebiotic and 1 × 10^7^ CFU L^-1^ *P. acidilactici *probiotic

The results of bacterial count in prebiotic and control treatments showed that the concentration of lactic acid bacteria in these treatments over various time of enrichment were at a level lower than 20 CFU g^-1^ and no significant difference (*p* > 0.05) was observed in these treatments at different hr of sampling.

The levels of bacteria in enriched treatments by pro-biotic and synbiotic were almost at the same level but with the passage of time after the start of enrichment, attached *P. acidilactici* to adult artemia had an increasing trend (Fig. 1). However, no statistically significant differences were observed between bacteria attached to adult artemia at 4 and 6 hr after start of enrichment (*p *> 0.05).

## Discussion

To the best knowledge of the authors, this study was the ﬁrst attempt to investigate the optimal enrichment *A. franciscana* with a synbiotic (*P. acidilactici* and FOS). Indeed, only a few studies have reported the effects of different probiotics enrichment in artemia on fish growth and survival. 

In the present study, bacterial levels used in the enrichment solutions at all sampling times were at a level equivalent to 10^10^ CFU g^-1^. Gomez-Gil *et al*. during enrichment experiment of *A. franciscana *with *Vibrio parahaemolyticus* and *Vibrio alginolyticus* applied the concentrations of 10^7^ CFU g^-1^ and 10^8^ CFU g^-1^, respectively, and reported that their changes at different times of the enrichment followed the same pattern. ^25^

Similar studies were not observed regarding to enrichment of adult artemia with probiotic and synbiotic. Therefore, all comparisons were made with enriched artemia nauplii. Concentration of attached bacteria to adult artemia, showed positive results with the passage of time. The same results by Parta *et al*. were obtained during enrichment of *A. franciscana* nauplii with yeast (*Saccharamyces baulardii*) with 24 hr after the enrichment and reported yeast in nauplii accumulated at a level equivalent to 3.5 × 10^3^ CFU g^-1^.^26^ However, enrichment experiments of *A. franciscana* nauplii with two strains of *Vibrio *showed different patterns, so that, attached bacteria to artemia nauplii began to increase at first 30 min after start of enrichment, then suddenly dropped at 8 hr after the enrichment and again a sharp rise occurred at 24 hr in levels of bacteria in nauplii which all Nauplii died at the end of this time.^25^ *Artemia urmiana* had a gradual trend in enrichment with mentioned probiotic bacilli that was added over time to attached bacteria. Campbell *et al*. in the enrichment of *A. franciscana*, with the formalin-killed of species *Vibrio angualiurum*, showed that when the concentration of bacterial suspension of the enrichment was 1.5 × 10^7^ CFU g^-1^, the maximum accumulation of attached vibrios to the artemia nauplii would happen at 60 min and at a concentration lower than that (1.5 × 10^6^ CFU g^-1^) at 120 min after the start of enrichment.^27^ Changes in the number of bacteria in the *A. franciscana* by the number of bacteria in *A. urmiana* nauplii was not limited by the number of bacteria in enrichment suspension and the same results were reported by Makridis *et al*. in the enrichment of *A. franciscana* nauplii with the probiotic bacteria.^17^

Results of this experiment indicated that adult artemia had high ability in enrichment with the probiotic bacteria *P. acidilactici* and enrichment time had a positive ratio with attached bacteria to artemia.
